# Enhanced Mental Health with Virtual Reality Mental Hygiene by a Veteran Suffering from PTSD

**DOI:** 10.1155/2021/5576233

**Published:** 2021-07-03

**Authors:** Bo Søndergaard Jensen, Niels Andersen, Jes Petersen, Lene Nyboe

**Affiliations:** ^1^Clinic for PTSD and Anxiety, Aarhus University Hospital Skejby, Denmark; ^2^DTU Space, Denmark; ^3^PrimeView Film Production, Denmark

## Abstract

This paper describes the application and feasibility of the use of Virtual Reality Mental Hygiene (VRMH) as a mean to reduce anxiety and stress in a Danish veteran suffering from posttraumatic stress disorder (PTSD) and enduring personality change after a catastrophic experience. The results from this case study provide preliminary evidence that VRMH can be used as a mean to reduce arousal in patients with severe PTSD.

## 1. Introduction

Posttraumatic stress disorder (PTSD) is a mental disorder that can occur after severe or prolonged mental trauma of an exceptionally threatening or catastrophic nature such as serious accidents, war, natural disasters, or rape. PTSD is characterized by reexperiencing the traumatic event in the form of invasive memories, flashbacks, and nightmares. The patient experiences severe anxiety when exposed to circumstances reminiscent of the traumatic event and avoidance of anything reminiscent of the trauma, both physically and mentally, is common. There are persistent symptoms of mental hypersensitivity and alertness characterized by difficulty falling asleep, irritability, difficulty concentrating, hypervigilance, and tendency to risk-seeking behaviour (e.g., driving recklessly, taking drugs, and having unprotected sex).

PTSD is often a relatively chronic disorder, and about 1% of the population suffers from PTSD at any given time. Among soldiers who have been at war, as well as victims after disasters, the incidence of PTSD is about 10% and among victims of rape about 30% [1, 2].

Exposure therapy as a treatment of PTSD has received significant support from many randomized controlled trials as well as meta-analyses. A Cochrane meta-analysis concluded that individual trauma-focused cognitive behavioural therapy (TFCBT), EMDR, stress management, and group TFCBT are effective in treating PTSD, whereas other nontrauma-focused psychological treatments do not reduce PTSD equally significantly [3].

Prolonged exposure therapy (PE), which is an individual TFCBT, has been shown to be effective in treating PTSD from various types of traumatic events including combat actions [4–6]. APA's clinical guidelines from 2017 as well as NICE guidelines from 2018 strongly recommend the use of PE in the treatment of PTSD [7, 8].

The dropout rate for treatment with PE is between 13% and 38% [9], and 20-50% of those who complete the treatment maintain their PTSD diagnosis despite significant symptom reduction [10–13]. So, even though the treatment of PTSD with PE has proved promising, there is still a need for further improvements in treating patients with PTSD. Therefore, there is a basis for investigating potential methods in addition to the existing treatment. One possible intervention could be Virtual Reality Mental Hygiene (VRMH) as a mean to reduce stress and anxiety.

In this case study, the project hypotheses were that VRMH could activate the soothing system and the parasympathetic nervous system in the patient, and this activation could have a positive impact on the general mental health and overall well-being.

The intervention manual was developed within the theoretical framework of compassion-focused therapy [14, 15]. The modern human brain is a product of a long evolution, in which different evolutionary conditions have formed the brain, with the essential purpose of survival. Different parts of the brain reflect different motivations, abilities, and interests, and this affects our experiences and the way we live our lives in different ways.

The less evolved part of the human brain, *the reptilian brain*, is concerned with territory, food, reproduction, and survival. The more evolved *mammalian brain* is concerned with living in groups, hierarchy, status, and caring. The most evolved part of the human brain is concerned with extended caregiving, attachment, and thinking.

These different parts of the brain are linked to our three major emotional regulation systems, *the threat system*, *the drive system*, and *the soothing system*. Each of the emotional regulation systems is associated with distinct states of feeling, motivations, behaviour, neuroanatomy, and neurochemistry. Healthy functioning requires adaptive use of all three systems in appropriate measures. Dysfunction can occur due to limited flexibility or overuse of one system to the detriment of others [16].

When the threat system is dominant, our mind is focused on seeking protection from dangers motivated for survival. Our emotions are about fear and anxiety, and our physiology is highly aroused with a behaviour dominated by fight or flight.

When the drive system is dominant, our mind is attuned towards wanting, seeking, aspiring, or striving. Our emotions are positive and motivated, and our physiology is aroused with a behaviour dominated by focus on specific goals.

When the soothing system is dominant, our mind is attended to giving and receiving care, affection, and nurturance. Our emotions are about safeness, and our physiology is calm with a behaviour dominated by looking-after and soothing.

The three emotional regulation systems are connected to the human autonomic nervous system. The threat system and drive system are mainly connected to the sympathetic nervous system, and the soothing system is mainly connected to the parasympathetic nervous system [16, 17].

The sympathetic nervous system is responsible for the fight or flight response. In the first moments after a stressor occurs, the hypothalamic pituitary adrenal axis (HPA axis) is stimulated. The HPA axis is responsible for the neuroendocrine adaptation component of the stress response. The response is characterized by secretion of cortisol, adrenaline, and noradrenaline. It increases pulse, breathing, and cardiovascular efficiency and dilates the bronchioles and pupils. It also divides fat into fatty acid and glycerol, breakdowns glycogen stored in liver to glucose, causes outflow of blood from the limbs to muscles, heart, and brain, and stops digestion. All this is to prepare the body for a fight or flight response.

At a certain blood concentration of cortisol, adrenaline, and noradrenalin, this protection response is achieved, and the there is a negative feedback to the HPA axis and systemic homeostasis returns. The parasympathetic nervous system is responsible for the body's rest and digestion response in which the body is relaxed, resting, or feeding. It basically undoes the work of sympathetic division after a stressful situation. The parasympathetic nervous system decreases respiration and heart rate and increases digestion.

Our sympathetic nervous system is designed to help us to survive in life-threatening emergencies, but if we spend too long in this heightened state of sympathetic activation, this can have negative consequences on our health. We will become exhausted, unsettled, experience cognitive decline, and experience poor sleep, and the immune system will be compromised. In the long run, this repeated exposure to stressors and activation of the sympathetic nervous system will also cause habituation of the organism to the stressor with repeated and sustained HPA activation. Therefore, it is important to support a healthy balance between sympathetic and parasympathetic activation.

One way to achieve this is through the activation of the soothing system and thereby activation of the parasympathetic nervous system. Our soothing system can be activated in various ways, e.g., through positive interaction with other humans, slow-breathing, mindfulness, yoga, body awareness therapy, positive self-talk/letter writing, and visualization exercises [16, 17].

## 2. Case Presentation

### 2.1. Participant

The patient included was a 45-year-old male Danish war veteran who had been in treatment for chronic PTSD and depression in the psychiatric outpatient clinic for PTSD and Anxiety at Aarhus University Hospital Skejby, Denmark.

Prior to his deployment to Bosnia in 1995 for a period of 6 months as part of the Danish UN mission, the patient was mentally inconspicuous. He describes the childhood home as safe, a place where he had received care and attention, and there were no problems with alcohol or violence in the family. He has generally had good relationships with other people and had few close friends. The patient had no mental health difficulties and had no mood swings or anxiety.

After the deployment, the patient was unemployed for about 7 months, before he got a new contact with the Armed Forces, where he served for a period of 4 years. After that, the patient had several shorter employments, from 4 months to 2 years, as an unskilled worker. Among other things, he was a factory worker, guard, doorman, slaughterhouse worker, and most recently a truck driver. He has been unemployed since 2012, and in 2019, he granted an early retirement benefit due to his mental health condition. After his deployment, he subsequently had an increase in mental health difficulties and a changed behaviour with mood swings, anxiety, and a pronounced tendency to distrust of other people. His mental problems had severe social consequences, partly in the form of a decline in his social network, periodic abuse of alcohol and drugs, and several times of disulfiram treatment of alcoholism.

Before the treatment at the Clinic for PTSD and Anxiety, the patient's PTSD had been treated with several different psychotropic medications, mainly SSRI and SNRI drugs. The patient sought treatment for the first time in 2001.

At intake to the clinic, the patient was unemployed and living alone. He had divorced his wife several years before. He had two children from this marriage but had little contact with them at the beginning of the treatment.

Initially in treatment, the patient described several psycho-traumatizing incidents from his deployment to Bosnia. These included war-related experiences such as shootings, explosions, fires, bombing, and being taken hostage and used as a bomb shield for several days.

In relation here to, the patient had repeated stressful intrusive memories of these experiences, e.g., nightmares dealing with incidents from his deployment. He did not describe any episodes of flashback or loss of reality. He became intensely emotionally upset when he was reminded of the traumatic events describing different directed emotions such as anxiety, anger, and sadness. The patient reported suffering from daily feelings of anxiety. He experienced discomfort when exposed to circumstances reminiscent of the trauma. The anxiety worsened when he was in larger gatherings of people and had panic anxiety accompanied by autonomic symptoms in the form of palpitations, chest tightness, and headaches. Together, the above symptoms led to a pronounced tendency to isolation. Thus, the symptom picture met the ICD-10 criteria for PTSD.

Firstly, the PTSD symptoms were treated with prolonged exposure therapy (PE) with a significant reduction of symptoms, especially the invasive symptoms. Although there was a significant reduction of symptom level, he still met the diagnostic criteria for PTSD. Specifically, his symptoms of hypervigilance were not changed. Further, he experienced a persistent change in personality.

After his deployment, he became significantly more distrustful and hostile to the outside world, had severe difficulties being with other people, and had chronically increased tension, as if he was always facing a threatening situation, even though he objectively knows well that the situation is not dangerous or that other people will not hurt him. He had a persistent feeling of emptiness and hopelessness and feelings of being alienated. Thus, there were clear signs of a personality change accordingly to WHO's ICD-10 diagnosis F62.0 enduring personality change after catastrophic experience.

The patient agreed to participate in this pilot study examining whether Virtual Reality Mental Hygiene (VRMH) could be a possible intervention to reduce stress and anxiety for patients with PTSD. He was reassured about confidentiality by emphasizing that information would not be made available to a third party without his permission, and that results would only be used for publication in professional journals. The patient gave informed consent for participation in the study, in accordance with the Helsinki Declaration.

### 2.2. Intervention

In this study, the VRMH stimulation comprised a naturalistic 10-minute virtual reality film of the beach by the west coast of Denmark with a 360-degree view. This was chosen in agreement with the patient as he had previously perceived this as a calming and soothing environment. The video was accompanied by the sound of waves from the ocean with the purpose of giving a more naturalistic experience, in all designed to activate the soothing system and the parasympathetic nervous system in the patient.

The VR headset used in the study was a pair of PIMAX 4K with 1000 Hz dual gyroscope with 18 ms MTP (motion to photons) and a 360-degree FOV (field of view). The PC had an Intel Core i5-7500 CPU with 3.40 GHz and 8.00 GB RAM. The graphics card was a GeForce GTX 1060 3 GB.

During two months of study, the patient was instructed to use VRMH every afternoon. Further, the patient was instructed to be aware of any side effects when using the VR glasses, e.g., vertigo. During the two months, the patient had weekly sessions with his psychologist giving the possibility to address side effects. Apart from vertigo and in rare occasions nausea, there are no known side effects of using VR. For this patient, VR was seen to be an easy and applicable treatment for addressing his persistent feelings of hypervigilance that had not improved sufficiently during treatment.

Before the study, the patient's PTSD and comorbid depression had been treated with prolonged exposure therapy and 200 mg of Sertraline with a significant reduction of PTSD symptoms. For a period of two months before and during the VRHM study, there were no changes in pharmacotherapy.

### 2.3. Assessment

Psychiatric status was assessed using the Mini-International Neuropsychiatric Interview (M.I.N.I.), which is a short structured diagnostic interview developed by psychiatrists and clinicians in the United States and Europe for DSM-IV and WHO's ICD-10 psychiatric disorders. The M.I.N.I is screening for 17 most common disorders in mental health according to DSM-IV and ICD-10.

The disorders investigated are the most important to identify in clinical and research settings.

For each disorder, one or two screening questions rule out the diagnosis when answered negatively.

It was designed to meet the need for a short but accurately structured psychiatric interview for multicenter clinical trials and epidemiology studies and has an administration time of approximately 15 minutes [18].

For measuring the effect before and after the use of VRMH, a self-report questionnaire was developed for assessing feelings of anxiety, stress, vigilance, anger, and sadness. On one side of the self-report questionnaire, the patient was instructed to mark on a 10 cm long visual analog scale (VAS) from zero to ten, where zero marked not at all and ten marked very much, how much the item bothered him, before the use of VRMH, and on the other side with an identical self-questionnaire, how much the item bothered him after the use of VRMH, without looking at the previous rating.

Differences between the assessment before and after VRMH were statistically analyzed using paired *t*-test, with a significance level of 0.05. All analyses were performed in SPSS ver.20.

## 3. Results

In all, the patient performed 40 daily assessments before and after use of VRMH, and the statistical analyses are based on these. Besides feelings of “stress,” all items showed statistically significant reductions from before to after use of VRMH (Table 1). The feeling of “stress” seemed to worsen after use of VHMR; however, this was not statistically significant.

## 4. Discussion

In this single case study, the use of VRMH significantly reduced feelings of anxiety, vigilance, anger, and sadness in a Danish war veteran with an ICD-10 diagnosis of PTSD (F43.1) and an ICD-10 diagnosis of enduring personality change after catastrophic experience (F62.0). The case study therefore provided preliminary evidence that VRMH can be useful in reducing symptoms of arousal and anxiety in a Danish veteran with chronic PTSD. However, the feeling of stress seemed to worsen after use of VRMH. A possible explanation for this could be that the item “feelings of stress” is not so clearly defined as other feelings being assessed. It could also be due to the fact that sitting quietly when wearing the VR equipment presents a genuine stress factor for this particular patient. Previously, VR has mainly been applied to patients with PTSD as an exposure to traumatic events, e.g., war [19]. In accordance with our results, a recent study of VR of nature videos improved feelings of anxiety and stress in patients with severe psychiatric disorders [20]. However, it cannot be ruled out that a large proportion of the intervention effect can be attributed to nonspecific factors and not to the specific method. Nonspecific factors such as the therapeutic alliance and perceived social support could have influenced the outcome more than the specific intervention.

Another limitation of the study is that there was no long-time follow-up with measurements of more psychiatric symptoms as, e.g., PTSD and depression.

The use of biological measurement during the use of VRMH could also strengthen the validity of the collected data. These could be measurements of galvanic skin response (GSR) or heart rate variability (HRV).

GSR is an autonomic respond in the sweat glands of the skin, controlled by the sympathetic nervous system (SNS), and the skin conductance is an indication of physical and psychological arousal. If the SNS is activated, the sweat gland activity is also activated, and higher arousal in the SNS also increases sweat gland activity and thereby increases the skin conductance; hence, GSR can be a measurement for emotional responses, and a clinical interview or questionnaire could verify what kind of emotion had caused the response [21].

HRV could be another biological maker for the effectiveness of VRMH stimulation to promote general mental health. HRV is the variation in the time interval between consecutive heartbeats, and HRV represents the ability of the heart to respond to different psychological and physiological stimuli in the environment. Low HRV is associated with impaired regulation of the ANS, and a reduced ability to cope with internal and external stressors and high-resting HRV has been associated with physical and mental health.

Neuroimaging studies suggested that HRV may be linked to cortical regions as the ventromedial prefrontal cortex (vmPFC) and the dorsolateral prefrontal cortex (dlPFC). The vmPFC is involved in stressful situation appraisal, and several studies have provided support for the important role that the vmPFC plays as a moderator and inhibitor of the amygdala.

The dual inhibition model posits that the vmPFC and the dlPFC, along with their associated inhibitory pathways, must interact for adequate inhibitory control of the amygdala and emotional regulation in PTSD [22, 23].

The reduction in this VRMH study of feelings of anxiety, vigilance, anger, and sadness could be attributed to a positive stabilization of the HRV, but this is still unclear and needs further study during the application of the VRMH.

Based on the above, VRMH could be an intervention for further investigation as mean to enhance general mental health by patients with chronic PTSD.

Clearly, results from a case study have limited generalization, and there is a need for further research into the use of VRMH. With the addition of biological measurements as GSR and HRV, it could be confirmed whether or not the perceived reduction in anxiety is a result of a decreased activation of the SNS. It would also be of interest if the VRMH has a lasting impact on the HRV, so follow-up studies with measurements of the HRV could strengthen the quality of future studies.

In addition, further research conducted in a general psychiatric clinic with outpatient care could strengthen the general evaluation regarding the utility and capacity of this intervention to both reduce psychopathology and promote general mental health under controlled conditions (efficacy) and to be successful when implemented in routine treatment settings (effectiveness).

## Figures and Tables

**Table 1:** Mean differences between before and after use of VRMH.

Item	Before (mean (SD))	After (mean (SD))	Mean difference (SD) (95% CI)	*p* value
Anxiety	3.54 (0.47)	3.37 (0.44)	0.17 (.43) (0.03; 0.31)	.02
Stress	3.57 (0.48)	3.50 (0.49)	0.06 (0.37) (-0.05; 0.18)	.26
Vigility	4.44 (0.63)	4.23 (0.68)	0.22 (0.48) (0.07; 0.38)	.006
Anger	3.72 (0.73)	3.45 (0.74)	0.27 (0.60 ) (0.08; 0.46)	.007
Sadness	3.52 (0.82)	3.23 (0.66)	0.29 (0.65) (0.08; 0.49)	.009

## Data Availability

Data is available on request through the first author.
